# Additive Manufacturing in Off-Site Construction: Review and Future Directions

**DOI:** 10.3390/buildings12010053

**Published:** 2022-01-06

**Authors:** Jubert Pasco, Zhen Lei, Clodualdo Aranas

**Affiliations:** 1 Department of Mechanical Engineering, University of New Brunswick, Fredericton, NB E3B 5A3, Canada; clod.aranas@unb.ca; 2 Off-Site Construction Research Centre (OCRC), Department of Civil Engineering, University of New Brunswick, Fredericton, NB E3B 5A3, Canada; zhen.lei@unb.ca

**Keywords:** additive manufacturing, off-site construction, Industry 4.0, smart manufacturing

## Abstract

Additive manufacturing (AM) is one of the pillars of Industry 4.0 to attain a circular economy. The process involves a layer-by-layer deposition of material from a computer-aided-design (CAD) model to form complex shapes. Fast prototyping and waste minimization are the main benefits of employing such a technique. AM technology is presently revolutionizing various industries such as electronics, biomedical, defense, and aerospace. Such technology can be complemented with standardized frameworks to attract industrial acceptance, such as in the construction industry. Off-site construction has the potential to improve construction efficiency by adopting AM. In this paper, the types of additive manufacturing processes were reviewed, with emphasis on applications in off-site construction. This information was complemented with a discussion on the types and mechanical properties of materials that can be produced using AM techniques, particularly metallic components. Strategies to assess cost and material considerations such as Production line Breakdown Structure (PBS) and Value Stream Mapping are highlighted. In addition, a comprehensive approach that evaluates the entire life cycle of the component was suggested when comparing AM techniques and conventional manufacturing options.

## 1. Introduction

The prospect of revamping our approach to modern construction using Additive manufacturing (AM) techniques is a promising but inherently cautious research field. Despite the rapid adoption of AM in multiple industries, such as the electronics, biomedical, defense, and aerospace sectors, available construction industry projects are mostly at the development stages [1,2,3]. Progressive countries are heavily exploring the option of automating construction techniques to cope with the limited availability of skilled labor and perform delicate building procedures [4]. The layer-upon-layer fabrication capability of AM, or 3-D printing, presents unique advantages in construction, such as streamlining of beam connections, optimized stress distribution in structural forms, reduced construction time, efficient energy utilization, reduced material waste, enhanced safety during construction, and seamless integration of printing parameters with building information modelling (BIM) [5,6,7]. The added complexity and automation do not necessarily require additional costs, unlike conventional construction techniques, which would almost always entail increased expenditures for intricate structures [7]. Structural parts can be accurately modeled and placed at optimal locations without the restriction of conventional construction shapes. Material usage can also be efficient without a compromise on the load-bearing capacity or other necessary properties. Since the construction industry has a considerable impact to the environment (being responsible for 36% of annual global energy use and 39% of energy-related carbon dioxide emissions [8]), adoption of AM technologies can also aid in carbon mitigation and provide other ecological benefits [9]. Changes in design can be accommodated late in the construction process, and parts can be reliably built inside or outside the construction site. The maintenance and replacement of damaged metallic parts are also possible for laser additive manufacturing techniques [10]. Certain AM techniques are also explored for rapid reconstruction of shelters in areas struck by unexpected calamities [11]. Furthermore, for the same material, unique mechanical properties can be attained using AM due to the fast cooling rates inherent in the laser printing process. Post-processing techniques such as heat treatment and thermally induced prestressing can further customize the resulting material properties [6]. The uncertainty associated with the structural performance of new components produced using AM is an essential research field due to the inherent risks associated with using construction components.

The types of feasible structures in AM are practically unrestricted even at a high resolution (such as angular structures, overhangs, internal openings, arches, beams, or cantilevers [7]). Still, some AM technologies are severely limited in terms of maximum component size. Currently, the maximum build size commercially available for sand molds is an AM system with a printing dimension of 4.0 × 2.0 × 1.0 m [12]. Another challenge for AM in construction is incorporating other systems that are necessary for buildings, such as insulation, piping, and electrical wiring. Some of the techniques to be introduced later readily accommodate additional materials during the fabrication process [1]. Besides this, components can be strongly anisotropic after fabrication, which will be detrimental to the load-bearing capacity and stress distribution in the resulting structure. Therefore, extensive analysis of the mechanical and geometric properties of AM printed materials is also necessary.

Several construction projects have explored the use of AM processes [6,13,14,15,16,17,18]. The idea of using AM technology in the construction industry is to build cheaper houses with minimal material waste and man-hours. However, despite the potential benefits, the adoption level of AM in the construction industry is significantly lower than in other private sectors [19].

**Figure 1:**
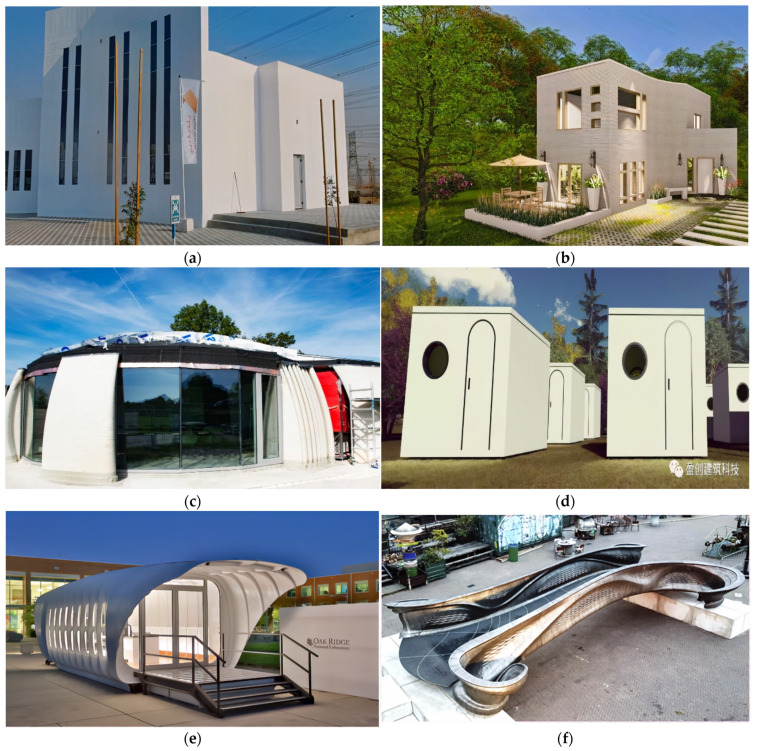
Recent AM Projects in the Construction Industry: (**a**) Apis Cor 3D printed building in Dubai [13] Copyright 2021 Copyright Apis Cor. (**b**) Apis Cor demo house in Louisiana [14] Copyright 2021 Copyright Apis Cor. (**c**) CyBe double-curved building in Netherlands [15] Copyright 2021 Copyright CyBe Construction. (**d**) Winsun smart temperature-measuring disinfection checkpoints [16] Copyright 2021 Copyright Yingchuang Building Technique (Shanghai) Co., Ltd. (WINSUN) (**e**) Additive Manufacturing Integrated Energy (AMIE) building in USA [17] (**f**) MX3D metal 3D-printed footbridge in The Netherlands [18] Copyright 2020 Copyright Elsevier Ltd.

In 2019, Apis Cor worked with the Dubai municipality to build the world’s largest 3D-printed building to date, which is presented in Figure 1a [13]. A mobile 3D printer was used to fabricate the 9.5 m high formwork for the two-story office wall structures using a gypsum-based mixture as printing material. Apis Cor also intends to procure the standard permits necessary for residential approval of a 3D-printed demo house in Jackson, LA, USA, as shown in Figure 1b [14]. The 3D-printed house structure was built in two weeks and is claimed to be more economical compared to masonry construction. On the other hand, CyBe produced intricate concrete walls on a double-curved building in the Netherlands, shown in Figure 1c [15]. As the first commercial building featuring 3D concrete printing in Europe, the AM technique used is claimed to produce lower overall CO_2_ emissions and reduced material waste. The Chinese company Winsun, which produced multiple house projects using 3D-printed components, proposed the creation of anti-epidemic prefabricated buildings such as smart temperature-measuring disinfection checkpoints as a response to the global pandemic in 2020, as shown in Figure 1d [16]. Large-scale structures made of thermoplastic composites can also be fabricated using an AM technique called Big Area Additive Manufacturing (BAAM), which is currently being researched at the Oak Ridge National Laboratory in USA using a large-volume 3D printer provided by Cincinnati Incorporated. The method features the possibility of zero-waste construction, as displayed in the Additive Manufacturing Integrated Energy (AMIE) building shown in Figure 1e [17]. Another notable construction AM project is the 2.5m-wide and 10m-long 3D-printed footbridge shown in Figure 1f [18]. MX3D, in collaboration with Arup engineers and researchers from the Imperial College, London, conducted full-scale structural modeling and testing to verify the load-bearing properties of the bridge [6]. The AM technique used was the Wire + Arc Additive Manufacturing (WAAM) technology, which will be further discussed later.

Meanwhile, the off-site construction approach has gained popularity in the past decades. Offsite construction comprises a wide range of construction projects with various definitions. By nature, off-site construction refers to the construction approach in which building elements are manufactured in places other than their final installation locations. There have been a series of different terms for this: panelized construction [20], prefabricated construction [21], modular construction [22,23,24], etc. In its evolution over the past decades, two strategies were implemented: the product and manufacturing concepts [25]. These concepts tend to treat the building elements as products that can be standardized for prefabrication. This has led to productivity increases and rising popularity in the construction industry. In the past decade, off-site construction has gradually become a cost-effective and productive construction approach for project delivery, compared to the stick-built construction approach. According to a report by McKinsey, modular construction can speed up construction projects by as much as 50% and potentially cut project costs by 20% [26]. Off-site construction has also gained popularity in cold regions and areas with a shortage of labor supplies. For example, in northern Alberta, Canada, heavy industrial construction relies on prefabricated modules that are delivered to the site for installation [27]. This helps eliminate the exposure of workers to extreme weather.

**Figure 2:**
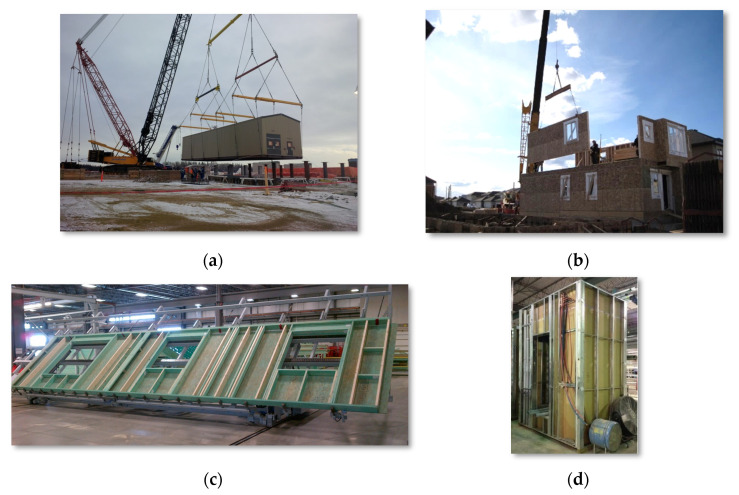
Typical off-site construction examples: (**a**) Heavy industrial module installation, (**b**) Wood panelized residential construction, (**c**) Wood panel manufacturing, and (**d**) Light-gauge-steel bathroom pods.

In a typical off-site construction context, the construction components are produced, prefabricated, and assembled in off-site facilities or open spaces, then shipped to construction sites for installation. In such a manner, the off-site production and on-site construction schedule overlap to yield project-cost savings and schedule shortening. Depending on the complexity and level of modularization, the off-site construction is categorized into a wide variety of products used in different construction sectors (see Figure 2 for typical off-site construction examples): (1) Panelized construction: this approach is often adopted in the building construction sector, where building projects are divided into individual wall panels/roof sections for manufacturing. Typical construction materials used in this type of construction approach are light gauge steel and wood [20]; (2) Modular construction: this approach is also known as volumetric construction units. Construction companies manufacture these units in off-site facilities and ship them to site for installation; and (3) other prefabricated modules: examples of such include industrial components, such as vessels and equipment skids.

The combination of rapid and cheaper construction, offered by off-site construction and AM technology, respectively, can be a game-changer in the construction industry if the applications can be accurately identified. Thus, the present review paper presents the types of methods and materials in AM, and the potential applications and integration of AM technology in both on-site and off-site construction industries.

## 2. Additive Manufacturing Processes in Construction

The potentials of AM technology allowed the development of an international standard (ISO/ASTM 52900) in 2015 to formally establish the terms used in AM. The ASTM standard differentiates AM technology from conventional manufacturing techniques such as machining, forging, rolling, casting, and extrusion. These traditional processes are also known as subtractive and formative manufacturing techniques. Prominent additive manufacturing techniques acknowledged to possess tremendous potential in transforming the construction industry are highlighted, particularly: (i) material extrusion, (ii) binder jetting, (iii) powder bed fusion, and (iv) directed-energy deposition. AM processes for cement and polymers are introduced, but the following discussion will focus on metallic structural applications.

### 2.1. Material Extrusion

Most of the existing research and applications of AM in the construction industry are focused on the concrete-extrusion research field. Various designs for components of the extrusion process, such as nozzles, printheads, hoppers, scrapers and positioning systems, have been introduced [11,28,29,30]. Printing parameters are optimized, such as the printing path, gantry speed, extrusion rate, and layer height [28]. Diverse concrete printing materials are also available, categorized in [31] as plain concrete, geopolymers, fiber reinforcement concrete, rapidly hardening materials, and earth-based materials. The characteristics of extruded materials are also usually analyzed based on the pumpability (facilitation of material delivery), printability (resolution of material deposition), buildability (load-bearing capacity and stability of material layers) and open time (acceptable tolerances of the aforementioned properties) [32]. The balance between printability and buildability appears to be a crucial aspect during printing, since instability during fabrication can induce zones of weakness in the extruded material [3]. Since multiple reviews have considered Contour Crafting and Concrete Printing as pioneer extrusion-based technologies in the construction industry [1,29,32,33,34], a general comparison will be provided. Nevertheless, other promising processes have emerged such as Digital Casting Systems [35], 6-axis robotic arm-based extrusion processes [29,30], Batiprint3DTM [36], CONPrint3D [37], and XtreeE [38], among several others.

#### 2.1.1. Contour Crafting

Contour Crafting (CC) is one of the extrusion-based technologies that have been extensively considered for large-scale on-site structural applications, patented in 2010 at the University of Southern California [11]. CC utilizes computer-controlled troweling as a scraping tool to smoothen the deposited surfaces of a cement-based paste. A large nozzle attached to a gantry system is used for extrusion, and the movement of the actuator can be controlled in any direction defined by the cartesian coordinates. The layer-by-layer approach allows mechanization for conduits of plumbing and electrical services and does not necessarily require external support structures during fabrication, although doors and windows still require manual construction [1]. Research in CC technology has focused on improving the geometrical accuracy, extrusion rate, linear speed, and understanding of material behavior during printing [11]. Multi-story structures are still improbable due to the gantry size limitation, but tool path optimization and the addition of stable anchor points to allow a climbing mechanism for the machine platform are explored [39]. The authors in [40] also listed the quantitative aspects of contour crafting, which usually needs less build time due to achieving a volumetric fabrication rate of 0.018 m^3^/h for a 55.7 m^3^ material volume.

#### 2.1.2. Concrete Printing

Concrete Printing is another extrusion-based method introduced by the University of Loughborough that focuses on specific construction components, such as panels and walls with functional voids [33], and not the entire architectural assembly in CC [32]. The printing speed is improved, but the usual trade-off is a lower printing resolution, so the layers can exhibit anisotropic mechanical properties [1]. Alternatively, post-processing and finishing techniques can be applied depending on the component requirements. The authors in [40] also listed the quantitative aspects of contour crafting, such as its typical volumetric fabrication rate of 0.090 m^3^/h for a 128 m^3^ material volume.

Compared to conventional concrete, 3D-printable cementitious materials exhibit unique properties such as rheology, printability, and compressive strength due to the nature of their processing requirements. Therefore, to establish the crucial design factors that dictate the eventual performance of 3D-printed concrete, a multi-level material design (MMD) approach was employed in [41], considering the three main stages associated with fabrication. The key stages are identified as mixture design, printing process, and composite structure.

Mixture design focuses on the selection of raw ingredients that will be incorporated into the feed material. A general classification of 3D-printable concrete was presented in [31] by grouping the 3D-printable concrete materials as plain concrete, geopolymers, fiber reinforcement concrete, rapidly hardening materials, and earth-based materials. Furthermore, admixtures and other materials that enhance the feed material’s properties should be considered, such as supplementary cementitious materials (SCM), superplasticizers, fibers, aggregates, and viscosity enhancement agents (VEA) [41,42].

The next stage to consider focuses on production and how the feed material selected will be delivered and deposited. Although multiple types of extrusion techniques can be employed during concrete printing, most approaches can be analyzed in terms of building material transportation, printhead mechanisms, subsequent material deformation during deposition, and material properties promptly after deposition [43]. The effects of gravity-induced flow, viscoplastic flow inside the system, tensile forces along the nozzle opening, and compression forces during deposition are assessed during the design stage by testing the characteristic requirements of the material, such as pumpability, extrudability, shape retention, printing open time, and buildability [32,41,43,44].

After appropriate material selection and material handling has been satisfied, the final factor to consider relates to the assessment of the material’s structural performance based on the resulting mechanical properties [41]. The concrete’s unique anisotropic properties and exposed interface bonding layers should be catered to the actual application, especially since the mechanical properties will be distinctive compared to conventionally produced concrete. Factors that influence the interlayer bond strength were enumerated in [42]. Researchers have shown the effects of printing parameters (such as the interval time window in [45]), material properties (such as thixotropy in [46] and yield strength), and the printing environment (such as curing condition, surface moisture, and admixture activity [47]). In order to improve the crack resistance of the resulting concrete structure under various kinds of loads, another option explored is the addition of reinforcements [41]. Components that boost the material ductility can be introduced either separately to the printing or simultaneously during printing. For instance, the Material Deposition Method (MDM) was used to directly integrate metal cables as reinforcements to 3D-printed concrete [48]. Bend tests have revealed comparable performance of reinforced 3D-printed concrete to conventional reinforcement in cast concrete, although further research was found necessary to prevent failure due to cable slip. Recent applications have also integrated topological optimization to ensure efficient material usage during the post-tensioning of a 3D-printed concrete girder [49].

#### 2.1.3. Fused Deposition Modelling

**Figure 3:**
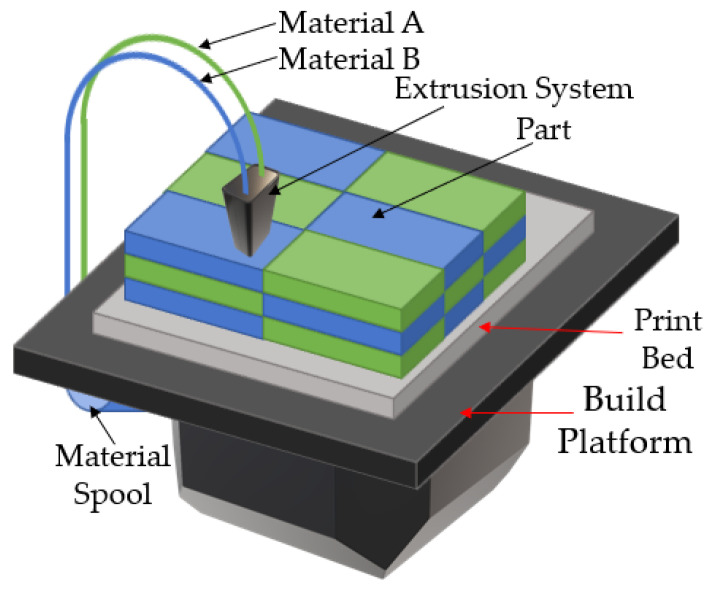
A diagram showing the fused-deposition process [50] Copyright 2016 Copyright Elsevier Ltd.

The schematic diagram of the fused-deposition technique is presented in Figure 3 [50]. The technology was initially introduced by using a polymer as a feedstock material due to its low melting temperature. In this method, a thermoplastic filament (such as acrylonitrile butadiene styrene or ABS, and polylactic acid or PLA) is fed into a heated extrusion nozzle. The applied heat converts the solid polymer into a semi-liquid state to allow the material to flow from the nozzle onto the print bed to form a thin layer. The print bed can be heated to decrease the cooling rate of the material. The process repeats layer-by-layer to fabricate the desired shape based on a computer-aided-design (CAD) model. The thermoplastic property of the polymer is critical to fuse the material during solidification while cooling. The layer thickness, width, temperature, printing orientation, and processing environment affect the quality of the build. In general, the final product has anisotropic properties. The mechanical properties (i.e., tensile and yield strengths) along the building direction are significantly weaker than along its transverse direction [50]. Thus, the strength of the interface between layers plays a major role in the bulk mechanical properties of the material. The anisotropic behavior of such materials limits their usage as a component for structural applications. The introduction of a fiber reinforcement attempted to solve such shortcomings. However, the bonding between the fiber and polymer matrix and void formation in the polymer matrix become additional issues. High-performance polymers, such as polyether ether ketone (PEEK), polyetherketoneketone (PEKK) and polyethylenimine (PEI) may also be employed in AM [51]. These polymers may have mechanical properties similar to those of conventional steel.

The fused-deposition process is not just limited to polymers; metallic wires can also be employed as a feedstock material to form complex geometries. This manufacturing process is known as Wire Arc Additive Manufacturing (WAAM) [52], similar to an arc-welding technique [53]. This technique has been around since 1925 [52]; however, significant progress has only been made in the past ten years. It can rapidly manufacture huge metal parts with high material utilization and low infrastructure cost. In this method, the metallic wire is melted and extruded onto a metal substrate, which acts as a bed. Since the process is somewhat similar to that of arc welding, the weldability of metals is critical. The alloys that have been studied so far using this technology are steels, titanium alloys, aluminum alloys, and nickel-based alloys [54]. The manufacturing time can be significantly reduced by 40–60% compared to conventional processes such as machining [55]. In the aerospace industry, it has been shown that aircraft landing gear ribs could be produced using WAAM (instead of traditional subtractive methods) with reduced raw materials by as much as 78% [53].

### 2.2. Particle Bed Processes

**Figure 4:**
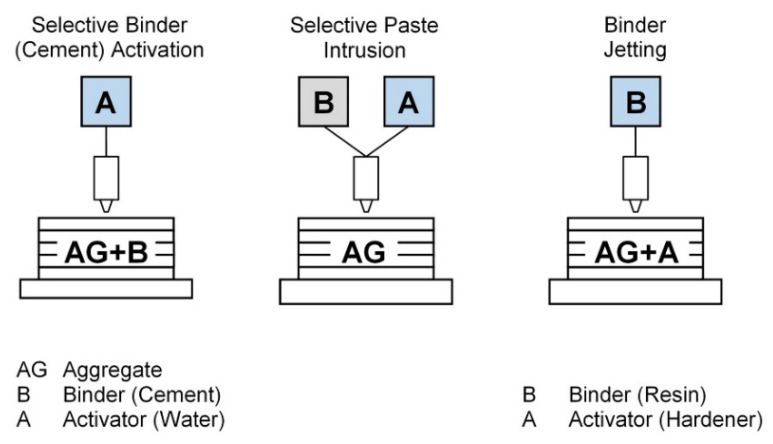
Particle bed AM processes in the construction industry [7] Copyright 2018 Copyright Elsevier Ltd.

In contrast to extrusion-based processes, particle-bed 3D concrete printing repetitively deposits layers of dry particles and a binding fluid phase using a print head or a nozzle. The process is followed by de-powdering and can further involve strengthening by infiltration or heat treatment [7]. As shown in Figure 4, the authors in [7] classified the techniques based on the order of the steps and the materials used. Selective binder activation locally bonds a dry mixture of cement and binder by introducing water or a water-admixture solution into the particle bed. On the other hand, selective paste intrusion starts with only the aggregates in the particle bed. A binder paste with cement, water and admixtures is then selectively deposited to fill the voids and attain the necessary component strength. Lastly, binder jetting consists of mixing the aggregates with a hardener component in the particle bed, after which a liquid binder is sprayed to react with the target components.

The use of particle-bed techniques that utilize concrete started with free-form construction ideas by Pegna that used different masks to selectively deposit and activate layers of Portland cement [56]. Larger components were then achieved using a method called D-shape^®^, a gantry-based off-site process with a 6 m-width particle-bed 3D-printer and a print head that contains up to 300 nozzles at 20 mm intervals [7,57]. Multiple passes are applied to ensure proper fluid bonding of sand layers with a thickness of 5 mm, distributed evenly by a blade. Surfaces are finished by sandblasting and polishing. Despite size limitations, the print head can already dispense either water, aqueous solutions, or cementitious slurries with limited geometric constraints. A high printing resolution involving 0.2 mm aggregate particles is feasible due to the particle-bed printing mechanism. The European Space Research and Technology Centre explored the construction of outposts on the moon using lunar soil processed by D-shape^®^ [57]. The technology has also been applied to various projects such as a footpath bridge in Madrid and the Radiolaria Pavilion [7].

#### 2.2.1. Binder Jetting

**Figure 5:**
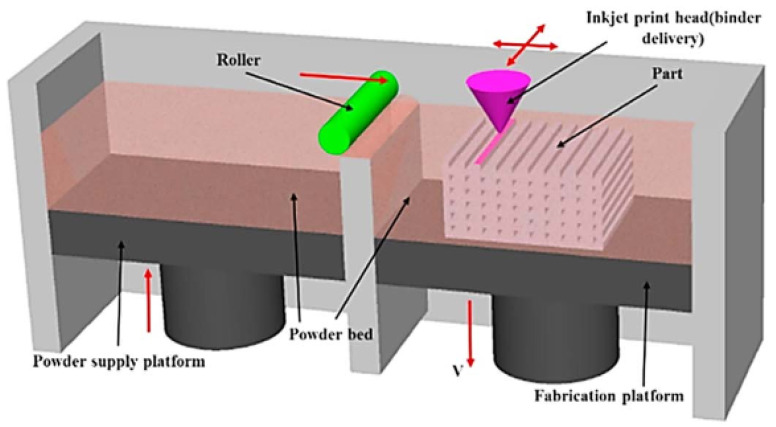
A schematic diagram of inkjet printing [50] Copyright 2016 Copyright Elsevier Ltd.

Binder jetting, also known as inkjet printing, is widely used for ceramic materials. The schematic of the process is displayed in Figure 5 [50]. A layer of powder material is initially transferred from the supply platform to the fabrication platform. Then, an injection nozzle pumps droplets of liquid binder, which solidifies and fuses the layer of powders. The process repeats itself until a part is formed. The injection nozzle can also pump a ceramic suspension (a combination of powder and a liquid binder) onto the fabrication bed, instead of binding the pre-deposited powder. The suspension then solidifies (usually through evaporation) and forms a solid part in a layer-by-layer fashion. The combination of the binder (typically a polymer) and the powder ceramic material generates a final material, which is a composite (not a pure ceramic). The conventional way of fusing ceramic materials is through powder metallurgy, which requires high temperature and pressure. In binder jetting, the cost of such operation is minimized; however, a binding matrix is introduced, which might limit the application of ceramics. Moreover, the fusion between the layers is another issue. The technique allows the production of complex forms and shapes without incurring additional costs or increasing the production time. Auxiliary support systems are also unnecessary when producing shapes with internal structures or cantilevering forms [58]. The parameters in the binder jetting process are the viscosity of the liquid or suspension, particle size distribution, solid content, nozzle size, extrusion rate, and speed of nozzle head [50].

#### 2.2.2. Powder Bed Fusion

Existing metallic AM research in the construction industry presents exciting opportunities for unique structural engineering applications, and preliminary work includes testing of the production processes and standard mechanical properties [1]. Customized metallic structures can possess favorable force and stress distributions that will withstand loading in high-energy locations, such as seismic areas [5]. Metallic AM applications in the construction industry primarily use the laser powder bed fusion (LPBF) and directed energy deposition (DED) techniques [59]. In the LPBF process, a focused power source, such as a laser or an electron beam, is used to selectively melt and combine layer upon layer of powders distributed on a build plate arranged by a sliced computer-aided design of the target component [60]. The inherently high cooling rates and smaller laser beam spot size translate to a high processing resolution and a refined component microstructure that is not achieved in conventional fabrication processes [61]. LPBF components can surpass both the tensile strength and ductility of conventionally manufactured counterparts and strengthening the material will not necessarily entail a brittle part [62]. The unmelted powder can be recycled and reused after printing, which makes LPBF suitable for expensive and resource-critical materials [63]. The range of printable material types for LPBF are also continuously expanding, and there is also growing interest in multi-material printing using LPBF [64]. The LPBF process can fabricate components with high density and complex geometries at higher accuracy, but a crucial limitation is the size of the working envelope that dictates the maximum size of the part. The current largest available build envelope of an LPBF printer is Adira’s Addcreator with a maximum printing size of 1000 mm × 1000 mm × 500 mm. Printing speed can also be slow if a higher printing resolution is desired [53]. Comparison of the mechanical properties of different steel structures manufactured using LPBF and considered for construction applications will be presented later.

### 2.3. Directed-Energy Deposition

On the other hand, in the DED processes, the feed material is simultaneously delivered and melted by a thermal source, which could either be a laser, electron beam or a plasma arc [59]. Coaxial nozzles are used to feed either metallic powder or wires, and wire feedstocks can be more economical compared to powder counterparts [64]. Since DED is not restricted by the build envelope that exists in LPBF, larger component sizes and faster deposition rates are easily achievable. However, this introduces geometric imperfections that deviate from the target model, such as surface roughness and lack of straightness in the components [65]. Therefore, mechanical and geometrical analysis of the printed parts is necessary. Post-processing techniques such as cutting, machining, and polishing are usually performed to smoothen the surface finish [64]. Since DED usually has a lower resolution than LPBF, present applications are limited to simple shapes such as cladding, and tasks such as repair and restoration of components. The inherent feeding system of DED also allows flexibility in utilizing multiple powder nozzles that can potentially be adapted for multi-material applications [66].

**Figure 6:**
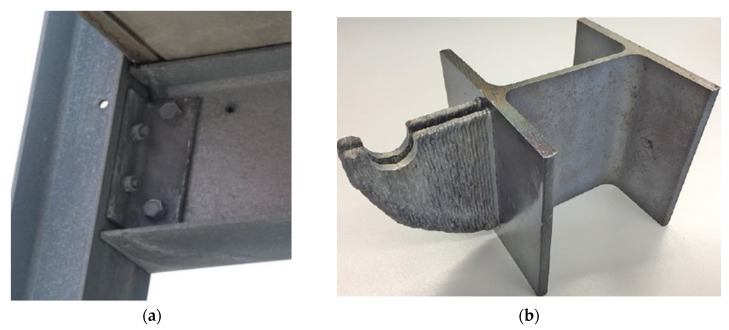

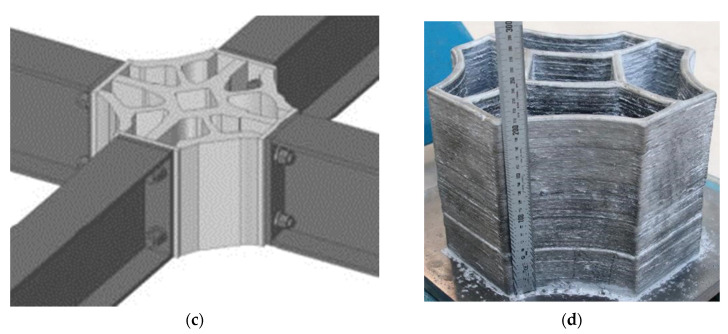
Topological Optimization of steel connections [67,68]: (**a**) Original bolt connection (**b**) AM-fabricated hook as replacement (**c**) 3D model of optimized node (**d**) AM-fabricated node that can connect four members. Copyright 2019 Copyright Ernst & Sohn Verlag für Architektur und technische Wissenschaften GmbH & Co. KG, Berlin; Copyright 2021 Copyright Ernst & Sohn Verlag für Architektur und technische Wissenschaften GmbH & Co. KG, Berlin.

A particular DED process that draws attention for construction applications is the Wire + Arc Additive Manufacturing (WAAM) technology, which utilizes heat from an electric arc to melt a welding metal wire feedstock on a motion assembly without theoretical dimensional limitations [54] To maximize the fabrication freedom inherent in AM, topology optimization of structural elements was explored for mild steel components fabricated using Wire Arc Additive Manufacturing (WAAM) [67,68]. Lightweight construction and efficient material utilization are achieved while maintaining the load-bearing capacity and rigidity of the overall structure. In addition to geometrical freedom, material inventory is reduced during construction since the storage of different plate sizes for subsequent connections is unnecessary. The optimization of a bolted flag plate replaced with a directly printable hook to distribute the stresses on a beam and column connection is presented in Figure 6a,b [67]. A customized node that can connect four steel beams, girders, or columns with symmetric loading is also shown in Figure 6c,d [68]. Other structural elements explored include I-section stiffeners that prevent the bending of a flange, clamps for braces that are erected at an angle, and T-stub end plates with a bolted connection. To demonstrate the topologically optimized components, on-site construction of a bridge was presented in Darmstadt [68]. Tensile tests show that the yield strength and tensile strength values were slightly below the material specifications for the mild steel filler wire used. Comparison of the mechanical properties of different steel structures manufactured using WAAM will also be presented later.

## 3. AM of Steel Structures in Construction Applications

### 3.1. State of the Art

In construction, the advantage of AM manifests from the benefits of using automation to assist in construction operations, removing labor-intensive repetitive tasks. Conventional construction techniques are restricted to repetitive shapes, rigid cross-sections of panels, permanent formworks, and interior structures. Beams and columns should be standardized in order to be affordable, so achieving optimal geometric shapes usually requires a higher cost for free-form fabrication [6]. Therefore, several applications of AM have been introduced in the past, including automating activities such as bricklaying, spraying concrete, milling, etc. [9,69,70]. The maturity of AM technologies in recent decades opens opportunities to advance the progress of automation in the manufacturing industry. In construction, research efforts have been directed primarily towards investigating how AM can be applied to specific construction tasks in the following areas: (1) algorithms and simulation development to assist AM implementations [71,72,73]; (2) design improvement of construction structures to enable AM implementations [74,75,76]; (3) innovative materials used for AM implementations to achieve higher printability [77]; (4) hardware to accommodate AM implementation in construction [78]; and (5) economics and scalability of AM implementation in construction [31,79,80]. Another motivation for AM applications is to achieve safety in harsh construction environments and to shrink the construction supply chain [2]. This leads to large potential benefits in the off-site construction industry, where construction work is preformed following a manufacturing style.

### 3.2. Wire Arc Additive Manufacturing

The Wire Arc Additive Manufacturing (WAAM) process is potentially scalable to large structural applications due to the capability of printing bulk amounts of metals with decent deposition rates, ranging from 1 kg/h to 4 kg/h for aluminum and steel respectively [53]. WAAM is being considered as an alternative fabrication method for parts such as solid billets and large forgings [18], but extensive testing of material properties, such as component load-bearing capacity and mechanical properties, is still necessary [34]. Existing research for WAAM has focused on titanium alloys, magnesium alloys, and aluminum alloys [81], but this section will focus on recent WAAM applications to steel alloys that have potential structural applications. Material tests on WAAM-manufactured 304L stainless steel [82], ER70S mild steel [54], HSLA steel [83], H13 steel [81], 308LSi stainless steel [65], and Low C-Mn steel [67] are investigated. A summary of the mechanical properties obtained from tensile tests of AM-printed metals compared to the required mechanical properties according to existing structural steel standards are listed in Table 1. Most of the values are averaged from multiple trials, but detailed test results are available in the references.

The WAAM process utilizes heat from an electric arc to melt a welding metal wire feedstock on a hardware assembly that usually includes a six-axis robot in a motion system, a power source, and clamping tools [54]. A computer numerical-controlled gantry system can also be operated to manage the multipass layer-by-layer motion. Increasing the deposition rate can compromise part integrity, so WAAM typically involves 1–2 mm single track layer heights that require finishing and machining after deposition. Some issues of WAAM are yet to be fully addressed, such as residual stresses from the high thermal input, mechanical anisotropy, standardization of techniques, low printing resolution, influence of as-built geometry, separation of material from the substrate, and the need for post-processing and product machining [54]. The authors of [53] suggested customized approaches to printing such as symmetrical building, part orientation optimization, and high pressure interpass rolling. Experimental methods were also developed to measure the cross-section shape irregularity, lack of straightness, and surface roughness of WAAM-fabricated components [65].

The wear, hardness, and tensile properties of WAAM-fabricated 304 stainless and ER70S mild steels were investigated [54]. Negligible porosity is observed, and tests show that the hardness, yield strength, and ultimate tensile strength properties of both the 304 SS and mild steel WAAM components are within the expected values of identical materials produced conventionally. Mechanical properties slightly improve, and wear rate decreases along the building direction, attributed to cyclic heating that induces annealing of subsequent layers [54].

The effect of heat input on the mechanical properties along different orientations of HSLA steels printed using WAAM were also investigated [83]. Nearly isotropic properties were observed after testing of hardness, tensile strength, and impact toughness of the vertical and horizontal samples. To control the temperature between layers and avoid excessive heat input, a constant dwell time was employed for cooling. In practical applications, this will, however, cause longer lead times for the process.

Different results were observed in H13 steel thin-walled parts fabricated by WAAM that use Metal-Inert Gas Welding (MIG) [81]. The part surface had minimal distortion, but the mechanical properties observed at different orientations with respect to the building direction were anisotropic. Hardness and ultimate tensile strength slightly increased in the upper layers along the building direction, while the middle layers attained the highest elongation values. Failure surface investigation also exhibited ductile fracture of all samples. Subsequently, a post-processing heat treatment at 830 °C for 4 h was applied to homogenize the resulting mechanical properties of the component. The hardness, yield strength and tensile strength values substantially decreased, but the total elongation increased, and the final components were isotropic.

Results from geometrical and mechanical property investigations of 308LSi austenitic stainless steel parts also presented the potential structural applications of WAAM [65,82]. The research was conducted at the Topography and Structural Engineering Labs of the University of Bologna in partnership with MX3D for the stainless steel bridge. The manufacturing process utilized a continuous printing strategy to fabricate different samples, such as planar sheets and full-scaled tubes, with a 1–3 mm layer height and an average printing speed of 0.5–2 kg/h. Geometrical characterization consists of digital calipers for measurements, analogic hydraulic scales for volume-based measurements, and a 3D-scanning system for cross-sectional analysis of one tubular element. Mechanical characterization consists of tensile tests, compression tests, buckling tests, and tensile tests with digital imaging correlation. A set of samples were also polished to obtain smooth surfaces and reduce the effect of geometrical inconsistency on the mechanical properties [82]. Tensile tests showed that averages of the yield stress, ultimate tensile stress, and elongation values are within the existing standards for structural components, although the Young’s modulus of elasticity value was lower than the expected value. Smoothed samples also exhibited isotropic behavior, but rough samples had different mechanical properties at different orientations due to geometrical inconsistency of the parts. Additionally, compression test results also exhibited identical elastic behavior compared to the tensile tests. Overall, polishing the samples improved the consistency of the material properties in different orientations with respect to the building direction [65].

### 3.3. Powder Bed Fusion

Available research on the structural performance of PBF-manufactured components, such as precipitation hardening steels [5], austenitic stainless steels [89], and high strength steels [59], usually involves customized test procedures such as tensile coupon tests, compression coupon tests, geometric imperfection measurements, and stub column tests. Results are then compared with the mechanical properties of conventionally produced components under existing standard provisions for structural applications such as the American Design Specification ANSI/AISC360 [85] and the European Code EN1993-1-1 [90].

**Figure 7:**
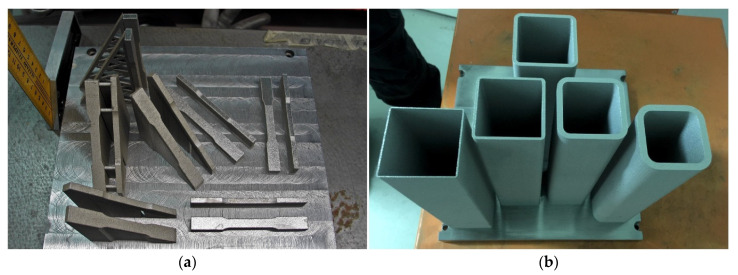
Printed Components for PBF Testing [5]: (**a**) PH1 specimens for tensile testing (**b**) 316L stub columns for compression testing. Reprinted with permission from ref. [5]. Copyright 2017 Copyright Elsevier Ltd.

Initial investigation of tensile and compressive mechanical properties of stainless steel PH1 and 316L fabricated using PBF in different orientations with respect to the building direction were performed and the printed components are shown in Figure 7a [5]. To assess the structural performance of the PBF-manufactured components, compressive properties of square hollow section (SHS) 316L stub columns were also compared to conventionally produced components. Processing parameters were maintained according to the manufacturer’s settings, and no discernible internal defects were observed in the printed components. Tensile PH1 and 316L samples with different orientations demonstrated identical Young’s modulus and proof stress values, but the values were lower than those of conventionally manufactured components. There is also significant variation in the ultimate tensile stress and fracture strain properties as the building orientation changes. An inverse relationship of the ultimate tensile stress and building angle was observed for PH1, but 316L samples did not show an obvious trend. The strength of the 316L samples was higher than conventionally manufactured components due to the fast cooling rates that occur during PBF [62], and appropriate heat treatment strategies were recommended to avoid the resulting material anisotropy for PH1 [91].

Compressive tests of the SHS 316L stub columns were also performed to assess the load carrying capacity of the PBF-manufactured components for structural applications, presented in Figure 6b. Results show that sufficiently thick stub columns reached substantial strain hardening before failure and demonstrated similar structural behavior with conventionally produced SHS stainless steel components according to existing standard structural performance parameters [5]. The importance of acquiring multiple test data that can be compiled and fitted into constitutive models to predict the deformation behavior of the components are also highlighted.

Similar tests were applied to examine the structural properties of H13 steel tubular sections printed using PBF with three different scanning patterns and four different sample orientations [59]. In addition, stub column tests also evaluated SHS columns, rectangular hollow sections (RHS), and circular hollow sections (CHS). Initial fabrication results showed excellent fabrication surface quality at all scanning patterns and sample orientations. Hardness values were within expected ranges, and high yield strength and ultimate tensile strength values were achieved. However, tensile tests revealed the poor ductility of the additively manufactured samples, which failed without necking at tensile strains of as low as 0.8–3.2%. Different tensile properties are also observed at various specimen orientations, suggesting that the samples are highly textured in the building direction. On the other hand, compressive tests of the stub columns showed similar properties at different scanning patterns and section shapes. All stub columns reached failure by material yielding, with inelastic local bucking at the ultimate load, which suggests that SHS, RHS and CHS structures are effective in compression. The study also compared the structural properties observed with existing standards in the American Specification, European Code, Australian Standards, and New Zealand Standards for steel structures and concluded that a design code specific to additively manufactured components is necessary.

## 4. Process Considerations

### 4.1. Input Considerations

#### 4.1.1. Cost Considerations

The impact of adopting AM technologies on the overall cost of construction is yet to be fully investigated, and multiple aspects of the AM construction process differ significantly from traditional construction techniques. Typical cost components, such as labor, material, and equipment, are affected differently if AM techniques are employed [9]. The accuracy of printing helps avoid remedial work and allows reasonable prediction of time and material expenditures. Automation can also reduce manpower requirements, but the benefits can be offset by the high cost of printing components. Procuring proprietary software packages also entail additional costs [92]. Challenges in introducing procedural changes and changing personnel roles should also be addressed. However, affordable 3D printers and technological familiarization will likely improve as adoption progresses in the industry.

**Figure 8:**
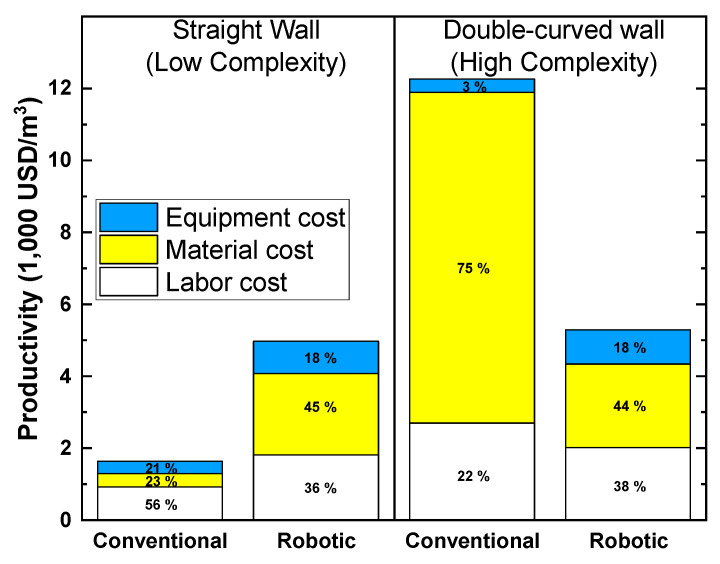
Breakdown of cost components in different construction scenarios. Reprinted with permission from ref. [9]. Copyright 2018 Copyright Elsevier Ltd.

The total cost and time requirements under multiple onsite construction scenarios of double-curved and straight concrete walls were investigated by comparing the use of automated AM techniques and conventional processes [9]. The project was conducted in the DFAB house in Dübendorf, Switzerland, and production data was sourced from onsite observations, contractor interviews, video recordings, and accepted literature. Results show that as the construction complexity increases, robotic fabrication becomes more economical compared to traditional techniques. Fabricating simple structures (such as the straight wall) did not yield cost benefits when using the automated techniques, but complex structures (such as the double-curved wall) were cheaper to produce. A breakdown of the cost allocation under different construction scenarios is also presented in Figure 8. The project expenditures were classified as either labor, material, or equipment costs. For assembling the simple wall, the productivity measured after using conventional techniques and automated techniques were 1639 USD/m^3^ and 5023 USD/m^3^, respectively. However, while constructing the double-curved wall worsened the productivity of conventional techniques to 12,262 USD/m^3^, the productivity of using automated techniques remained consistent (5288 USD/m^3^). Moreover, conventional construction cost allocations are highly varied depending on the complexity of the project. For instance, material cost allocation abruptly increased from 23 to 75% after switching from straight wall to double-curved wall construction. However, in automated fabrication, the cost breakdown is similar regardless of the complexity, and the cost allocation is more balanced (remained ~35:45:20 ratio between equipment, materials, and labor costs) compared to conventional construction. Although not presented, the entailed time savings and work breakdown advantages of automation observed in the case study also appear to be beneficial to the sustainability of the process.

#### 4.1.2. Material Considerations

**Figure 9:**
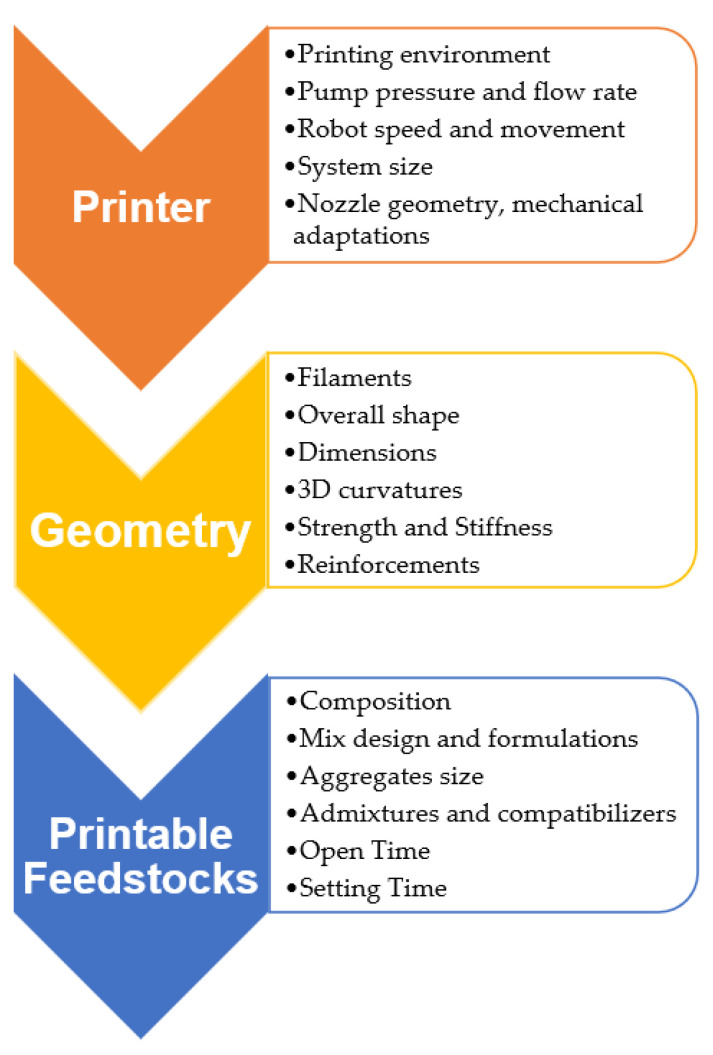
Material parameters considered for AM in construction [93].

As shown in previous sections, a significant portion of research concerning AM in construction focuses on investigating new material types, properties, and geometries possible to exploit the fabrication freedom offered by the technology. A wide range of process parameters interdependently influence manufacturing decisions in multiple stages of the construction process, and typical factors that affect material choice are presented in Figure 9. Based on the review article [93], the type of printer, product geometry, and suitable raw material mix should be considered in sequence. The target mechanical properties should reflect on the choice of feedstock composition, percentage of additives, and feed aggregate sizes. Material delivery should also be optimized in terms of the expected open time and setting time of the material blend. The pressure and flowrate of the printer should then be synchronized to the optimum feed blend. A balance between printing speed and dimensional accuracy should be attained considering the printer’s capabilities, and deposition complications such as feedstock hardening inside the printer should be avoided. Finally, target geometries should be integrated with the two previous parameters to breakdown the target structure in a layer-by-layer manner. Building blocks should be produced considering the target mechanical properties and produce stable shapes to attain actual structural properties that are close to the target building model [93].

### 4.2. Existing and Suggested Frameworks for Successful AM Implementation

#### 4.2.1. Performance Testing of Cementitious Blends

**Figure 10:**
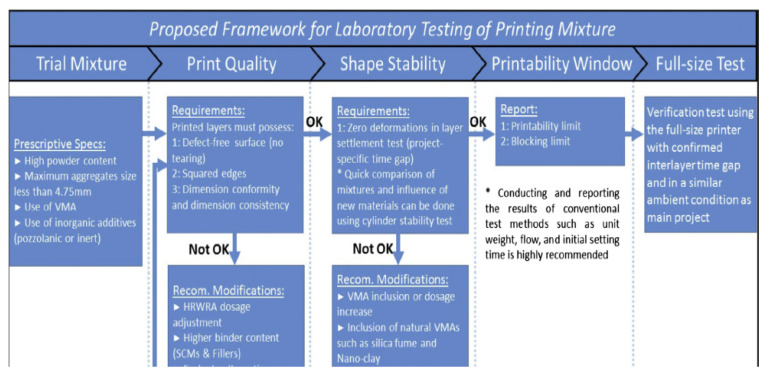
Proposed framework for performance testing of cementitious mixtures. Reprinted with permission from ref. [94]. Copyright 2018 Copyright Elsevier B.V.

The next step in improving the industrial acceptance of AM processes in construction is the development of frameworks and standardized guidelines that meet the requirements in conventional structural regulations. For instance, a framework that defines the acceptance criteria for testing different compositions of cementitious blends with potential construction applications has been proposed [94]. The proposed method, presented in Figure 10, is based on the assessment of fresh properties of blends, such as the print quality, shape stability, and printability window. Initial mixture properties such as powder content, aggregate size, paste fraction, and use of viscosity-modifying admixtures (VMA) should be considered. The resulting print quality of the composition should first be tested iteratively until the surface condition, dimensional consistency, and continuity of edges are at acceptable levels. Adjusting the mixture proportion or choosing an entirely new composition is proposed if the proposed benchmark cannot be achieved. Afterwards, the shape stability should then be evaluated based on successive tests such as the cylinder stability test and the layer settlement experiment [44,94,95]. The cylinder stability tests investigate the influence of different materials on the geometrical stability of the product, while the layer settlement experiment evaluates the stability of successive printed layers. No evident deformations in the resulting product should be observed. The other key property to be evaluated is the printability window, which refers to the maximum amount of time within which the blend can be deposited and printed with sufficient quality and without the occurrence of hardening or blockage in the nozzle. The effectiveness of the qualified blend mixture should then be verified in a full-size printer and the printing environment should be modified to mimic the ambient temperature and humidity of the actual construction project. The proposed framework was applied to four different mixtures and aided in comparing the performance of different blend proportions [94].

Performance tests that focus on the effect of material rheology should also be considered to refine the choice of printing materials and admixtures. For instance, the balance between pumpability and buildability of the material during printing should be determined. As was introduced earlier, pumpability refers to the ease of material transport through a pipe using pressure without encountering blockage issues that result in printing discontinuity and deteriorating material performance [41]. The shear viscosity of the material can typically be used as an indicator of material pumpability [96]. Multiple studies that relate pumpability and material rheology have been performed, but standard test specifications are still lacking [44]. On the other hand, buildability pertains to the dimensional consistency of the material during deposition despite experiencing constant stress when layers are repeatedly printed on top of each other [32]. Measuring buildability often relates to assessment of the material’s green strength, and various tests and analytical models have been developed for such applications [97]. Overall, a good combination of rheological properties is desired since high workability (good pumpability) is necessary during the pumping phase, but a low workability (related to good buildability) is preferred during material extrusion [98].

Furthermore, the resulting physical properties of hardened concrete should be equally assessed and designed based on the actual application. Although compressive and flexural tests used for conventionally produced concrete can also be adapted, an important distinction is in the existing anisotropy and interlayer bonding properties of 3D-printed concrete. Mechanical properties along three orientations relative to the building direction should be evaluated [44]. The importance of specimen preparation that also differs from conventional samples is highlighted in [42], and the most commonly applied test methods for 3D-printed concrete were identified as the direct tensile test [99] and splitting test [100]. Other test methods, such as the wedge splitting test, slant shear method, torsion bond test and shear strength test were also reviewed in [101]. However, universally applied standards are still yet to be fully developed [42] and the integration of such test procedures into the overall structural design framework should be pursued. In-situ monitoring tests are being explored, and [102] discussed the use of power consumption measurements, extrusion pressure measurements, electrical resistivity measurements, and computer vision for better quality control during printing have been discussed. Post-printing tests typically applied to metal samples can also be adopted, such as microscopy techniques and chemical characterization (e.g., XRD, EDS, and EPMA) to analyze the micro- and nano-level interactions that contribute to the hardened concrete’s resulting mechanical properties.

#### 4.2.2. Production Line Breakdown Structure and Value Stream Mapping

**Figure 11:**
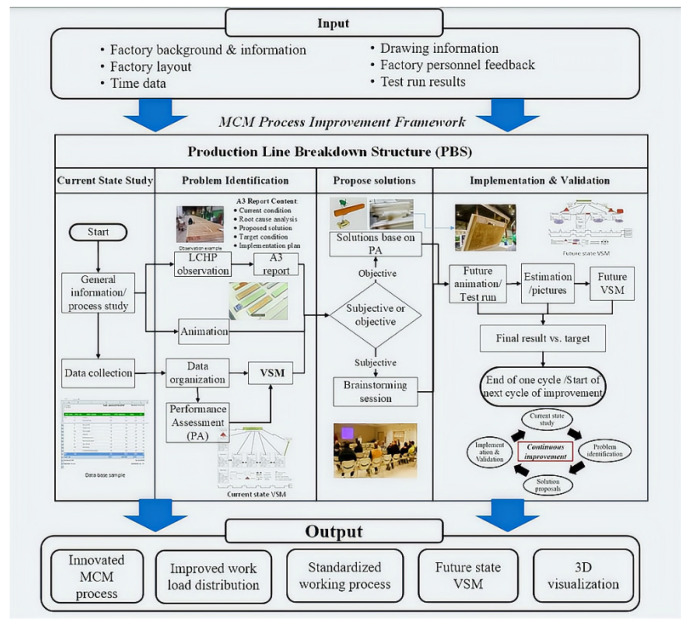
Proposed framework for improving the modular construction process. Reprinted with permission from ref. [103]. Copyright 2020 Copyright American Society of Civil Engineers.

The type of machinery and systems involved in AM are appropriate to offsite construction since the deposition system can be relocated from an outdoor to an indoor environment. On the other hand, a framework which combines key aspects of the construction process, the efficient approach of the manufacturing production line, and the concept of lean production principles to economically approach modular and off-site construction manufacturing (MOCM) was proposed and is presented in Figure 11 [103]. The initial step is process analysis and problem investigation using the production line breakdown structure (PBS). PBS is used to establish five different levels of the production plant with increasing complexity and degree of specificity. PBS can also be more applicable to an AM plant compared to other fundamental approaches such as work breakdown structure (WBS) since the workstations vary in size and focusing on the process rather than the deliverables will be appropriate. The same rationale was considered with MOCM, which was the focus of the framework.

Furthermore, lean manufacturing techniques were incorporated into the results of the PBS in the framework, utilizing value stream mapping (VSM) and key performance indicators (KPIs) in particular. VSM is used to diagnose and recommend opportunities for process enhancement in the production line condition by analyzing the flow of material and information exchange. VSM utilizes performance assessment concepts such as takt time, cycle time, lead time, and workhours. In addition, preceding activities and parallel activities should be considered in MOCM. Finally, the target KPIs and relevant components identified in VSM will then be used to gauge the appropriate changes for the current and future production line process. The proposed framework was then applied to a modular construction line at a MOCM facility in New Brunswick Canada, resulting in a 20% lead time reduction and 15% total work-hour savings. Prefabrication can therefore introduce labor cost reductions, efficiency in work management, better quality control, and flexibility in workspace requirements [103].

### 4.3. Potential Impact of AM to Sustainability in Processing

The potential of AM to significantly curtail resource and energy requirements has been recognized in low material volume, highly tailored, and resource-critical industrial fields, such as in the aerospace, medical, and tooling industries [104]. Utilizing the unique capability of AM to produce complex geometries such as topologically optimized structures [49,67] that are impossible to produce in any other way can result in better material efficiency and resource savings that are not attainable using conventional processes. Quantitative and qualitative sustainability evaluations in [104] further showed that, by 2025, AM can beneficially impact the manufacturing process through reducing production costs by 170–593 billion USD, reducing energy requirements by 2.54–9.30 EJ, and reducing associated CO_2_ emissions by 130.5–525.5 Mt. The overall reduction is around 5% for each sustainability criterion. A radical shift in licensing, patent, trademark, and copyright frameworks is also expected when AM technologies are adopted and integrated with existing construction regulations. Sustainability policies that therefore focus on technology, labor and regulatory frameworks were also recommended [104].

#### Life Cycle Analysis

Accordingly, Life Cycle Analysis (LCA) is a fundamental assessment tool that can complement the suggested frameworks and highlight the beneficial environmental impact of using AM in construction. Since multiple LCAs have been performed recently to investigate the suitability of using AM in building large-scale structures, the review in [105] consolidated trends and diagnosed the shortcomings in the available literature. The global warming potential (GWP) and Embodied Energy (EE) values of the published data for AM structures were directly compared to conventionally manufactured counterparts, such as aircraft parts, concrete floors, roof structures, brick façades, injection molds, and gas turbine system parts. GWP and EE values for AM were divided directly by the conventionally manufactured counterpart, and a threshold value was assigned as the basis wherein the values are assumed proportionate. Numerical results show the favorable environmental impact of AM in most cases, with most GWP and EE ratios being skewed towards the left of the threshold line, i.e., using AM results to lower GWP and EE value. Material usage was also found to be a major contributor to the GWP and EE values, both in AM and conventional processes. As previously discussed, the efficiency in material usage and the potential for recycling unused material in AM technologies will be a crucial selling point for the construction sector. The review also elaborated on the critical methodological errors that needed to be addressed for future LCAs, including choosing the correct functional units, defining system boundaries, citing sources of data, calculating LCA results using standard equations, and reporting uncertainty in and sensitivity of the results [105].

## 5. Conclusions and Recommended Research Pathways

**Figure 12:**
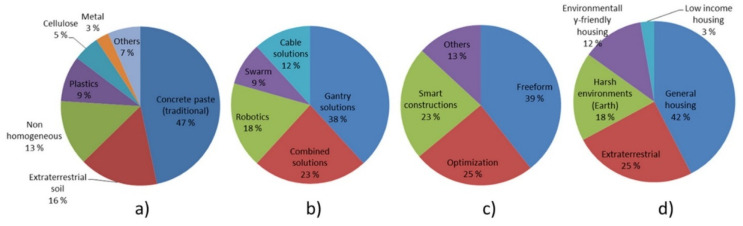
Publications of AM in construction sorted according to number of publications for (**a**) material science, (**b**) engineering, (**c**) architecture, (**d**) relevant markets. Reprinted with permission from ref. [3]. Copyright 2016 Copyright Elsevier B.V.

A systematic mapping study of publications exploring the potential applications of AM technologies in the construction industry was performed in [3]. The articles were categorized according to the number of publications for material science, engineering, building design, and market analysis, and a breakdown is presented in Figure 12. Publications that discuss concrete characterization and gantry solutions are prevalent, showing the lack of discussion concerning metallic AM structures that have potential construction applications. However, recent efforts in systematically analyzing the material properties obtained from AM techniques that are particularly suitable for metallic components, such as WAAM and PBF presented in this review, can pave the way for the industrial adoption of these techniques. The level of adoption should further improve when the range of printable metals in such AM techniques is expanded and properly characterized. Overcoming the size/resolution limitations that currently hinder printing of large-scale structures will be possible if technological innovations are driven by industrial interest. Identifying unique structural challenges that require optimized stress distributions and efficient material usage can drive interest in adopting metallic AM structures, especially if the target mechanical properties cannot be achieved using conventional techniques.

In the context of offsite construction, the option of prefabrication and offsite facility assembly fits most AM techniques since material fabrication can be performed in a controlled environment. Process automation and the dimensional accuracy assured in printable structures of AM techniques will be crucial in offsite construction if the components are to be assembled again at the actual construction site. Research should further involve cost and material considerations during printing, and the studies presented highlight the inherent advantages of AM techniques. In addition, the existing research should be integrated with standardized frameworks to attract industrial acceptance and conform to the requirements of conventional structural regulations. Strict standards are expected in structural load-bearing elements for buildings and large-scale structures. Finally, a comprehensive approach that also considers the entire life cycle of the structural product and the fabrication process should be employed when comparing the AM techniques to conventional manufacturing options.

## Figures and Tables

**Table 1:** Mechanical properties of metallic AM components.

AM Printed Metal	Manufacturing Technique	UTS (MPa)	YS (MPa)	Elongation
H13 Tool Steel	Conventional [84]	1990	1650	9%
	WAAM [81]	1085		10%
	WAAM + Heat treatment [81]	535.6	311.6	17.20%
	PBF [84]	1712	1236	4.10%
	PBF + preheating [84]	1965	1073	3.70%
308LSi SS	ASCE Standard [85]	586.1–620.6	248.2–275.8	
	WAAM (rough) [65]	569	347	33%
	WAAM (polished) [65]	521	303	22%
304 SS	ASCE Standard [85]	517.1	206.9	40%
	PBF (Building Direction) [86]	710	520	38%
	PBF (Transverse Direction) [86]	580	450	58%
316 L SS	ASCE Standard [85]	517.1	206.9	40%
	PBF (BD) [87]	579	439	21%
	PBF (TD) [87]	585	445	21%
	PBF (HIP + AHT, BD) [87]	592	257	47%
	PBF (HIP + AHT, TD) [87]	611	263	48%
15-5 PH SS	PBF (TD) [5]	1262	848	15%
	PBF (BD) [5]	1197	854	4.9%
ER70S Mild Steel	WAAM (BD) [54]	265	480	
	WAAM (TD) [54]	256	475	
	Wrought ASTM A36 [88]	247.5	400–550	
HSLA	WAAM [83]	700–795		17.1–25.6%
	HSLA ASTM A242 [88]	482.6	344.7	18–21%

## Data Availability

Data sharing not applicable.
